# Biostimulation of Petroleum-Contaminated Soil Using Organic and Inorganic Amendments

**DOI:** 10.3390/plants12030431

**Published:** 2023-01-17

**Authors:** Ogochukwu A. Udume, Gideon O. Abu, Herbert O. Stanley, Ijeoma F. Vincent-Akpu, Yusuf Momoh, Michael O. Eze

**Affiliations:** 1 Department of Microbiology, Faculty of Science, University of Port Harcourt, Port Harcourt 500004, Rivers State, Nigeria; 2 Department of Animal and Environmental Biology, Faculty of Science, University of Port Harcourt, Port Harcourt 500004, Rivers State, Nigeria; 3 Department of Environmental Engineering, Faculty of Engineering, University of Port Harcourt, Port Harcourt 500004, Rivers State, Nigeria; 4 Department of Genomic and Applied Microbiology and Göttingen Genomics Laboratory, Georg-August University of Göttingen, 37077 Göttingen, Germany; 5 Bioinstrumentation and BioMEMS Laboratory, Department of Mechanical and Aerospace Engineering, University of California, Davis, CA 95616, USA

**Keywords:** biostimulation, water hyacinth compost, spent mushroom compost, NPK fertilizer, bioremediation, TPH, priority PAHs

## Abstract

The most common approaches for the in-situ bioremediation of contaminated sites worldwide are bioaugmentation and biostimulation. Biostimulation has often proved more effective for chronically contaminated sites. This study examined the effectiveness of optimized water hyacinth compost in comparison with other organic and inorganic amendments for the remediation of crude oil-polluted soils. Water hyacinth was found to be rich in nutrients necessary to stimulate microbial growth and activity. An organic geochemical analysis revealed that all amendments in this study increased total petroleum hydrocarbon (TPH) biodegradation by ≥75% within 56 days, with the greatest biodegradation (93%) occurring in sterilized soil inoculated with optimized water hyacinth compost. This was followed by polluted soil amended with a combination of spent mushroom and water hyacinth composts (SMC + WH), which recorded a TPH biodegradation of 89%. Soil amendment using the inorganic fertilizer NPK (20:10:10) resulted in 86% TPH biodegradation. On the other hand, control samples (natural attenuation) recorded only 4% degradation. A molecular analysis of residual polycyclic aromatic hydrocarbons (PAHs) showed that the 16 PAHs designated by the US EPA as priority pollutants were either completely or highly degraded in the combined treatment (SMC + WH), indicating the potential of this amendment for the environmental remediation of soils contaminated with recalcitrant organic pollutants.

## 1. Introduction

Population growth, accompanied with an increasing need for energy, has led to the continuous exploration and extraction of crude oil [1]. Over the years, the increased crude oil output has had a severe unintended impact on aquatic [2,3] and terrestrial lives [4,5]. Crude oil is emitted into the environment in a variety of ways during drilling, transportation, and distribution [6,7]. These spills have detrimental effects on the ecosystem. Attempts to eliminate crude oil pollution from the environment have resulted in the creation of a variety of remediation methods [8]. Bioremediation, a method that employs living organisms—particularly microbes—to break down pollutants in a contaminated environment, has been the subject of intense research in recent decades [9,10,11,12,13,14]. Bioremediation (including its many variants such as phytoremediation) has been recognized as the most appropriate ecofriendly and cost-effective strategy for the cleanup of crude oil-contaminated habitats [15,16,17].

Bioremediation can either be carried out ex situ or in situ. As the name suggests, ex situ involves the excavation and translocation of polluted soil for off-site treatment [8]. Consequently, this method is very expensive and environmentally unfriendly. On the other hand, in situ technologies involve contaminant treatment on site, thereby making it an ecofriendly choice, since it has a minimal impact on the soil matrix and associated biota [8]. Typically, organic pollutants are treated in situ, and the most common approaches for the in situ bioremediation of accidental spills and chronically contaminated sites worldwide are bioaugmentation and biostimulation [18].

Bioaugmentation is the intentional supplementation of a polluted site with contaminant-specific degrading microorganisms [18]. This approach increases the number of degraders, thus speeding up the biodegradation of contaminants [19]. These degraders are obtained from contaminated environments (where they are accustomed to degrading contaminants even at high levels), cultured in a large quantity using target contaminants as the sole carbon and energy sources, and preserved for easy application. In view of the specificity of bioaugmentation and its resulting high number of microbial cells, it is often employed on sites requiring rapid cleanup, or sites that have recently been contaminated with a spill or have high concentrations of metallic and organic toxicants [20]. On the other hand, biostimulation involves the addition of nutrients or fertilizers (often referred to as amendments) to stimulate the growth and metabolic activity of indigenous microbes. There is generally a scarcity of nitrogen and phosphorus in the majority of polluted sites, limiting the ability of indigenous microorganisms to obtain sufficient nutrients for their survival and degradative activities. Adding rate-limiting nutrients to the system increases the degradation capacity of the microbial population that is already present. Sites suitable for biostimulation include those without an urgent need for clean-up, those moderate concentrations of contaminants, those with a long history of contamination, or those in a confined polluted aquifer [20].

While bioaugmentation has the advantage of speeding up the remediation of fresh spills requiring urgent cleanup, its major drawback lies in the inability of exogenous bacteria to thrive amongst indigenous microbes [21,22,23]. In addition, after a long period has passed following an oil spill, most of the volatile components of crude oil will have evaporated, leaving behind aged and weathered petroleum hydrocarbons that are often resistant to biological attack [24,25]. As a result, biostimulation has often proved more effective than bioaugmentation for the remediation of aged organic contaminants, requiring no urgent intervention [26,27]. Nutrients employed as biostimulants include organic and inorganic fertilizers. Macci, Doni [28], however, found that organic fertilizer was more effective in increasing soil organic matter content and microbial activity than inorganic fertilizer. Similar findings have been made by other researchers showing that organic nutrients are more likely to be released into the environment slowly and for a longer period, and with a beneficial impact on the soil microbiome [29,30,31]. For pollutant cleanup, biostimulation with either organic nutrients or with a combination of organic and inorganic nutrients has been proven to be more productive because it not only provides nutrients to microbes, but also enriches the soil[32]. Thus, organic fertilizers may be an ecologically safer source of nutrients for the biostimulation of petroleum-contaminated environments. One potential source of biomass for biostimulation is the aquatic plant *Eichhornia crassipes* (water hyacinth).

Water hyacinth is an invasive plant that has gained significant national and international attention due to its threat to the aquatic ecosystem [33,34]. Although native to South America, this plant has quickly spread to many parts of the world. Hence, it is a readily available source of biomass for composting purposes. Composting is an aerobic process that utilizes microbial activity to convert biodegradable materials into stable organic fertilizers that can be utilized as soil supplements, soil conditioner, or as biostimulants for contaminant biodegradation [35,36]. In view of the different circumstances requiring biostimulation and, in some cases, a combination of biostimulation and bioaugmentation, coupled with the rising need for ecofriendly and cost-effective biostimulants, the goal of this study was to examine the effectiveness of an optimized water hyacinth compost and/or spent mushroom compost to stimulate the biodegradation of crude oil-polluted soils. A lignocellulose-degrading bacteria isolate identified in Udume, Abu [37] as *Chitinophaga terrae* was used for the composting of water hyacinth. The resulting compost was then employed for the biostimulation of crude oil degradation. The results of this study revealed that water hyacinth compost is an effective soil amendment for the rapid biodegradation and remediation of total petroleum hydrocarbons and polycyclic aromatic hydrocarbons.

## 2. Results

### 2.1. Chemical Constituents of Water Hyacinth

The result of the chemical analysis of water hyacinth revealed that the plant is rich in potassium, with contents of approximately 6.5 ± 0.3%, 8.7 ± 0.4%, and 12.9 ± 0.4% in the root, stem, and leaves of the dried plant (Table 1). The nitrogen content of the dry plant is 2.0 ± 0.1%, 2.3 ± 0.1%, and 2.8 ± 0.2% for the root, stem, and leaves, respectively. The dried plant’s phosphorus content is 4.8 ± 0.2%, 5.3 ± 0.2%, and 7.1 ± 0.2% for the root, stem, and leaves, respectively.

The dry plant’s lignin content is 4.9 ± 0.1% (*w*/*w*), 4.8 ± 0.2% (*w*/*w*), and 3.6 ± 0.1% (*w*/*w*) for the root, stem, and leaves, respectively, while the cellulose content is 32.5 ± 0.3% (*w*/*w*), 30.2 ± 0.4% (*w*/*w*), and 27.3 ± 0.5% (*w*/*w*) for the root, stem, and leaves, respectively.

### 2.2. Enrichment and Isolation of Lignocellulose-Degrading Bacteria

The results of the enrichment of lignocellulose-degrading organisms using mineral salt medium (MSM) followed by enumeration on agar plates established the presence of microbiological function. An average value of 2.92 ± 0.10 × 10^6^ cfu/g of organisms was obtained for the samples on the fifth day (Table 2).

In addition, eight isolates were taken and examined for their cellulase-producing ability using carboxyl methyl cellulose (CMC) agar plates. The diameter of the halo zones—also known as the zone of clearance (a differentiated zone surrounding a central zone)—generated from the lignocellulose degradation screening shows the efficacy of the isolate to degrade lignocellulose in the water hyacinth. The results revealed that isolates B1, B4, and B7 are cellulase producers (Table 3).

### 2.3. Hydrocarbon-Degrading Potentials of Bacterial Isolates

**Figure 1:**
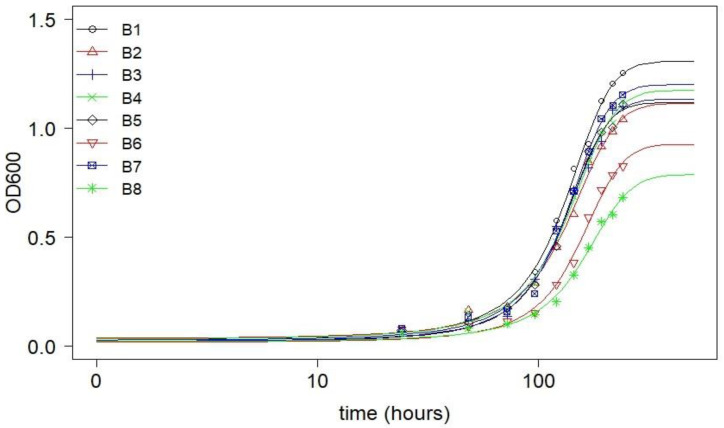
Three-parameter logistic model showing differential growth rates (in terms of optical density, OD600) of the bacterial isolates in crude oil-spiked liquid mineral medium.

During the initial two days of the trial (lag phase), there was no discernible alteration in the turbidity. However, a significant increase in the OD_600_ value was observed between the second and tenth days, indicating bacterial growth and metabolic activities (Figure 1). Based on the cellulase-producing and hydrocarbon-degrading properties of the microbes, isolate B1 was selected for the composting of water hyacinth biomass, following a series of composting optimization processes. The results of the optimization process were published in Udume, Abu [37].

### 2.4. Effect of Biostimulation on Crude Oil Degradation

**Figure 2:**
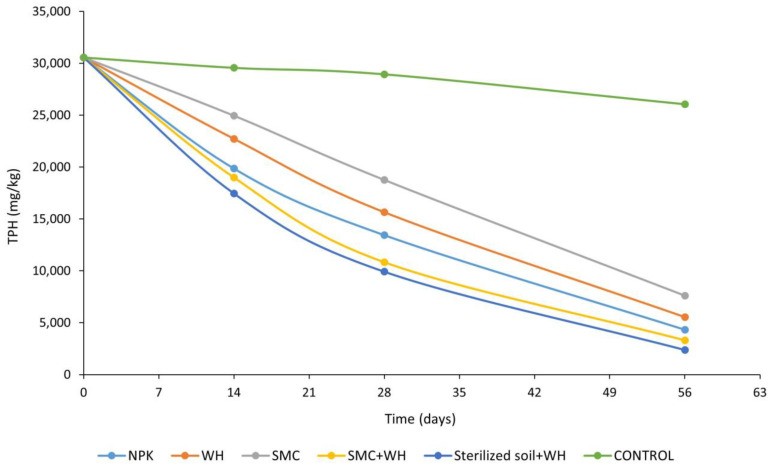
Total petroleum hydrocarbon dissipation in soils under different treatments during the experimental period. (WH: water hyacinth compost; SMC: spent mushroom compost; SMC + WH: spent mushroom compost + water hyacinth compost).

The bioremediation ability of composted water hyacinth biomass was compared with those of other biostimulants such as NPK fertilizer and spent mushroom compost. The preliminary investigation of the crude oil-polluted soil recorded 30,560.0 ± 6.9 mg/kg and 21,161.9 ± 9.2 mg/kg as the baseline value of total petroleum hydrocarbons (TPH) and polycyclic aromatic hydrocarbons (PAH), respectively (Figure 2).

The geochemical analysis of residual hydrocarbons revealed that sterilized soil amended with optimized water hyacinth (WH) had the greatest TPH reduction from 30,560.0 ± 6.9 mg/kg to 2371.7 ± 2.6 mg/kg, representing 93% biodegradation (Figure 2). This was followed by polluted soil amended with a combination of SMC and WH composts, which recorded a residual TPH level of 3297.3 ± 2.0 mg/kg. The unsterilized polluted soil samples treated with water hyacinth compost witnessed 82% TPH degradation to 5543.2 ± 3.2 mg/kg, while the residual TPH levels in polluted soils amended with spent mushroom compost were 7593.4 ± 5.6 mg/kg. Inorganic amendment using NPK fertilizer led to an 86% TPH reduction from 30,560.0 ± 6.9 mg/kg to 4313.2 ± 3.1 mg/kg. On the other hand, the control samples witnessed only a 4% TPH degradation from 30,560.0 ± 6.9 mg/kg to 29,297.3 ± 6.2 mg/kg at the end of the 56-day remediation period.

**Figure 3:**
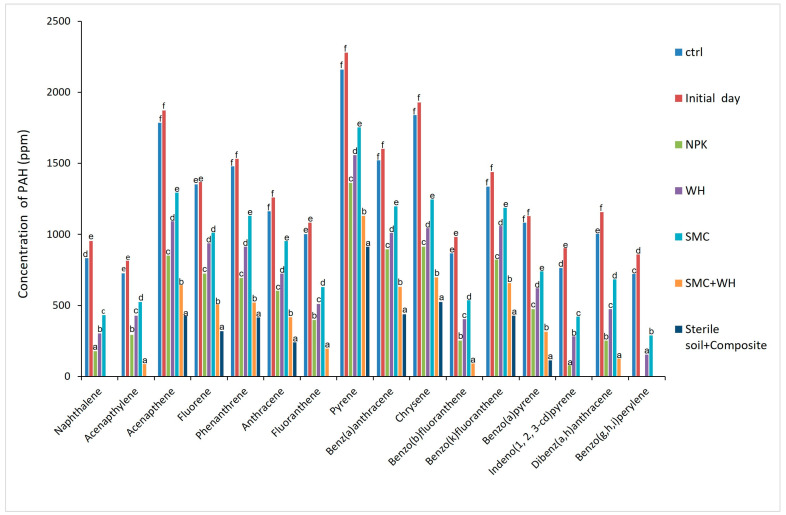
Residual polycyclic aromatic hydrocarbons in soils at the end of the experimental period under different treatments. (WH: water hyacinth compost; SMC: spent mushroom compost; SMC + WH: spent mushroom compost + water hyacinth compost). Bars within the same compound group but with different superscripts denote significantly different treatments (*p* < 0.05).

The molecular analysis of residual polycyclic aromatic hydrocarbons revealed that, like TPH degradation, the greatest dissipation of PAH occurred in sterilized soil inoculated with optimized water hyacinth compost (Figure 3). However, among unsterilized soil treatments, the combination of spent mushroom and water hyacinth composts had the greatest effect on hydrocarbon degradation, followed by the inorganic fertilizer, NPK.

### 2.5. Degradation Kinetics for TPH and PAH

**Figure 4:**
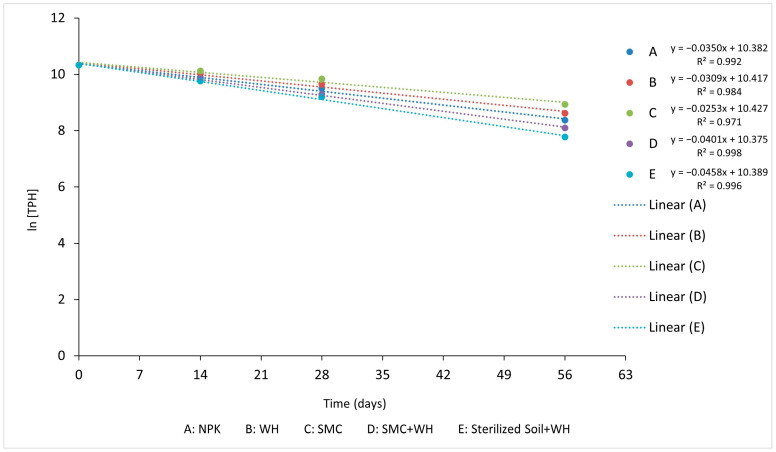
Plot of ln [TPH] versus time (days) showing degradation kinetic for the different soil amendments.

A plot of the natural log of TPH versus time under NPK amendment gave a linear kinetic equation of y=−0.0350x+10.382 (Figure 4, Table 4). Setup containing water hyacinth (WH), spent mushroom compost (SMC), spent mushroom compost + water hyacinth compost (SMC + WH), and sterilized soil + water hyacinth compost (Sterilized Soil + WH) amendments gave kinetic equations of y=−0.0309x+10.417, y=−0.0253x+10.427, y=−0.0401x+10.375, and y=−0.0458x+10.389, respectively. The R^2^ values, often referred to as “goodness of fit” [38], were all close to 1 (Table 4), indicating the fitness, replicability, and predictability of the derived model.

## 3. Discussion

Composting enables the transformation of biodegradable materials into stable humus-like substances that can serve as biofertilizers for soil amendments [39]. In this way, a range of biological wastes, such as livestock excrement, municipal solid trash, and industrial garbage can be harnessed for various agricultural and biotechnological applications [40,41], and the process is environmentally friendly, cost effective, and with minimal mechanical complexity [41].

Nitrogen, phosphorus, potassium, total organic carbon, and total organic matter contents were all found to be highest in leaves, while the roots had the highest levels of lignin and cellulose. The level of cellulose and hemicellulose were found to be several orders of magnitude greater than that of lignin (Table 1). This observation agrees with previous studies of water hyacinth [42,43,44,45]. These nutrients are vital for stimulating microbial growth and activity. This is especially important since microbial activity, among other processes, is the primary contributor to biodegradation [46]. These organisms depend on nutrients from plant biomass, such as organic acids, amino acids, and soluble sugars, for their nutritional needs and increased metabolic activities [47,48]. Composts rich in nitrogen, phosphorus, and potassium have been found to effectively enrich the soil, stimulate the growth of soil microbiota, and consequently rehabilitate damaged soils [49]. The stem had the highest values of reducing sugars and total carbohydrates, which are critical to microbial activities during composting and contaminant biodegradation [50]. Previous studies have revealed that plant biomass and plant-derived metabolites can alter microbial community composition and diversity in contaminated soils, leading to a shift in metabolic activities [28,29,51,52].

Microbial enrichment and enumeration on agar plates revealed that the highest average bacterial count occurred on the fifth day (Table 2). The continuous reduction in bacteria count after five days is an indication that the enrichment process aided in screening out organisms that were mostly unable to utilize water hyacinth as the sole carbon and energy source. The diameter of halo zones (also called the zone of clearance) generated from the lignocellulose degradation screening indicates that some of the isolates were capable of degrading lignocellulose in the water hyacinth (Table 3), with isolate B1 exhibiting the greatest cellulase-producing ability. The formation of the halo zone is an indication of the ability of bacteria to produce certain enzymes [53], and the occurrence of such halo zones on the CMC agar plate is the identifying property of cellulase producers [54,55]. Similarly, the hydrocarbon-metabolizing potentials of the isolates, evident from the OD_600_ values (Figure 1) revealed that isolate B1 is a potent petroleum degrader, indicative of its potential wider application for both composting and hydrocarbon biodegradation. The initial lag phase observed among the isolates corresponded to the time needed for the cells to adapt to their new environment in preparation for the exponential growth that would follow [56,57]. This process could include the repair of macromolecular damage that accumulated during the stationary phase [58,59]. Although this study was targeted at biostimulation, the survival and proliferation of microbes in the presence of a target contaminant is essential for the selection of organisms that may have wider application for bioaugmented remediation. Several studies have shown that petroleum hydrocarbons are toxic to living organisms, including plants [60] and microbes [61], and at high levels they may strongly inhibit bacterial growth and even lead to bacteria death [62,63]. On the other hand, several studies revealed a positive correlation between microbial population and petroleum degradation [64,65]. Hence, the need exists for organisms with the right metabolic potential for hydrocarbon remediation.

The organic geochemical analysis revealed the following order of biostimulatory effect for TPH biodegradation: Sterilized soil + WH (93%) > SMC + WH (89%) > NPK fertilizer (86%) > WH (82%) > SMC (75%) > Control (4%) (Figure 2). A similar observation was made for polycyclic aromatic hydrocarbons (Figure 3), indicating that the water hyacinth composting optimization process [37] was beneficial for the enhanced remediation of crude oil-contaminated soils. The fact that non-sterilized treatments experienced less hydrocarbon degradation than the sterilized soil + WH treatment indicates that the microbes used for composting might have a hydrocarbon-degrading ability but might not have effectively thrived against potentially less-efficient indigenous microbes in the non-sterilized soil. On the other hand, in the absence of indigenous microbes competing for nutrients in the sterilized soil, the compost-associated hydrocarbon-degrading microbes thrived, leading to enhanced biodegradation of crude oil hydrocarbons. A comparison of the non-sterilized treatments, however, enabled us to assess the differential stimulatory effects of the different treatments.

An important result from this study is the observation that a combination of different composted organic materials may prove more effective for the remediation of environmental pollutants. While the inorganic fertilizer NPK was more effective as a biostimulant than either spent mushroom compost or water hyacinth compost individually, the combination of spent mushroom and water hyacinth composts exerted greater biostimulation of crude oil degradation than NPK. In a similar study of organic and inorganic fertilizers, Macci, Doni [28] found that organic fertilizer was more effective in increasing soil organic matter content and microbial activity, and was, thus, regarded as vital for the preservation of soil quality and for the rehabilitation of degraded soils. Similar findings were made by Liu, Rong [29], who found that organic amendments produced more favorable effects on soil productivity and nutrient availability than inorganic amendments. The surprisingly low level of biodegradation (4%) occurring in the control treatment (natural attenuation) may be explained by the chronic nature of pollution at the sampling site.

Sixteen polycyclic aromatic hydrocarbons designated by the US EPA as priority pollutants (naphthalene, acenaphthylene, acenaphthene, fluorene, phenanthrene, anthracene, fluoranthene, pyrene, benzo[a]anthracene, chrysene, benzo[b]fluoranthene, benzo[k]fluoranthene, benzo[a]pyrene, dibenzo[a,h]anthracene, benzo[g,h,i]perylene, and indeno [1,2,3-cd]pyrene) [4,66,67] were either completely or highly degraded in the SMC + WH treatment, indicating the potential of this amendment (SMC + WH) for the environmental remediation of soils contaminated with recalcitrant and carcinogenic polycyclic aromatic hydrocarbons. Biodegradation kinetics provided information about the efficacy of various nutrient supplements in soil. The TPH in the set up amended with the NPK had about 86% degradation and a half-life of approximately 19.80 days, and a degradation constant of 0.035 (d-1) (Table 4). The half-life and degradation percentage of the different amendments supported the conclusion that the combination of spent mushroom and water hyacinth composts had the greatest stimulatory effect on biodegradation.

## 4. Materials and Methods

### 4.1. Water Hyacinth Collection

Fresh water hyacinth plants were manually harvested into canoes from the Orashi River located within Ahoada-East LGA, Rivers State, Nigeria. The plants were air-dried and fragmented with a shredder to improve ease of handling during composting. Spent mushroom compost was obtained from the agricultural farm at the University of Port Harcourt, Rivers State, Nigeria.

### 4.2. Enrichment and Isolation of Lignin-Degrading Bacteria

Lignocellulose-degrading organisms were taken from the rumen content of cows slaughtered at an abattoir in Choba community market, Obio-Akpor LGA, Rivers State, Nigeria, and enriched using mineral salt medium (MSM). One gram of sterile powder of water hyacinth biomass, acting as the source of carbon, was aseptically transferred into a 100 mL capacity Erlenmeyer flask containing 98 mL of MSM composed of 3 g/L of NaNO_3_, 0.5 g/L of KCl, 1 g/L of KH_2_PO_4_, and 10 g/L and 0.5 g/L of 7M anhydrous FeSO_4_ and MgSO_4_, respectively, adjusted to pH 6.3 [37]. The MSM was sterilized for 15 min at 120 °C, cooled, and seeded with 1 mL of rumen juice. For 20 days, the MSM broth seeded with rumen juice was agitated on a benchtop shaker at 120 rpm and 37 °C. At 5-day intervals, 100 µL of the culture was taken, serially diluted, and plated on carboxyl methyl cellulose (CMC) agar plates. This was to monitor the enrichment process through enumeration and consequently select the most viable isolates.

### 4.3. Screening of Cellulase-Producing and Hydrocarbon-Utilizing Potentials of Isolates

The purified isolates were evaluated for their capacity to degrade cellulose [68] following the method of Banerjee, Maiti [54]. In brief, the isolates’ cellulolytic activity was determined by flooding the methyl cellulose agar plates with Congo red to determine the halo zone. After 15 min, the plates were rinsed with 1M of NaCl, and the zones of clearance were determined [69]. The formation of a halo zone (a differentiated zone surrounding a central zone) is an indication of the ability of bacteria to produce certain enzymes [53]. The occurrence of halo zones on CMC agar plate is the identifying property of cellulase producers [54,55]. Subcultures of the desired organisms were prepared and maintained in slants for subsequent analysis. The approach outlined above was used to isolate bacteria capable of digesting cellulose from decaying water hyacinth.

The isolates were evaluated for their hydrocarbon-utilizing ability using the methods of Eze, Thiel [70]. In brief, a 1 mL (approximate) subsample of the purified isolate was added to an Erlenmeyer flask (300 mL) containing 100 mL of liquid mineral medium. Mineral medium was composed of KH_2_PO_4_ (0.5 g/L), NaCl (0.5 g/L), and NH_4_Cl (0.5 g/L). Sterile-filtered trace elements (1 mL/L) [71], vitamin solution (1 mL/L) [71], and MgSO_4_⋅7H_2_O (5 mL of a 100 mg/mL) were added to the mineral medium after autoclaving. One milliliter of crude oil was added to the flask as the sole carbon and energy source. The culture was grown at 30 °C on a benchtop shaker at 120 rpm for 10 days. During the 10-day period, bacterial growth in terms of optical density at 600 nm (OD_600_) was monitored every 24 h using the HachRDR2500 spectrophotometer (Loveland, CO, USA). Based on the OD_600_ values, the relative growth rates of the isolates were determined. 16S rRNA gene sequencing led to the identification of the most effective lignocellulose-degrading bacteria isolate (B1) as *Chitinophaga terrae* [37]. This isolate was then used for composting water hyacinth.

### 4.4. Composting of Water Hyacinth Biomass and Biostimulation of Crude Oil-Polluted Soils

The composting of water hyacinth and the optimization process were described in Udume, Abu [37]. In brief, this was carried out using seventeen 10 L capacity reactors kept in an open space. The reactors were supported on a raised platform. An equal amount of water hyacinth biomass was added to the reactors. The composting setups were inoculated with isolate B1 (*Chitinophaga terrae*). Compost operating parameters such as percentage moisture content, frequency of aeration by turning, and microbial seeding were maintained according to the experimental design. One gram of compost was drawn from each reactor after 21 days for lab investigation to determine lignin degradation.

The crude oil-polluted soil used for the remediation study was collected from a polluted site in K/Dere in Gokana LGA, Rivers State, Nigeria. Six pot-based treatment setups were performed in triplicates, namely: (1) polluted soil + NPK fertilizer; (2) polluted soil + water hyacinth compost (WH); (3) polluted soil + spent mushroom compost (SMC); (4) polluted soil + spent mushroom compost + water hyacinth compost (SMC + WH); (5) sterilized polluted soil + water hyacinth compost (SS + WH); and (6) unamended polluted soil (Control). The compositions of each pot were 1000 g of polluted soil amended with 10% (*w*/*w*) of either the organic compost or the inorganic fertilizer (Table 5). The entire experiment lasted for 56 days.

### 4.5. Organic Geochemical Analysis of Biodegradation

At the end of the experimental period, soils in each setup were homogenized following the method of Eze, Thiel [72], with some modifications. The following modifications were made: diethyl ether was used as the extraction solvent and extraction was based on the principle of mechanical shaking [73,74]. In brief, the soils were freeze-dried, and 5 g of the ground freeze-dried soil was further homogenized with a small amount of sodium sulfate (Na_2_SO_4_) and extracted using diethyl ether. Five grams of homogenized soil sample was weighed into a 100 mL beaker and 25 mL of diethyl ether added. The beakers were covered and placed in a shaker for 30 min. The extract was filtered through Whatman filter paper. The extraction process using diethyl ether was repeated twice, and the extracts were combined and then concentrated to a minimal volume using a rotary evaporator. Solid-phase extraction using a silica gel column was performed to remove fatty acids from the extracts. Residual total petroleum hydrocarbons and polycyclic aromatic hydrocarbons were determined using an Agilent 7890 GC system coupled with a 5975C VL MSD with a Triple-Axis detector gas (Agilent Technologies, Santa Clara, CA, USA). The extracts were transferred splitless to the GC column at an injector temperature of 300 °C. Helium was used as the carrier gas at a flow rate of 1.5 mL/min. The GC temperature program was as follows: 80 °C (hold 1 min), 80 °C to 320 °C at 5 °C/min (hold 20 min).

### 4.6. Biodegradation Kinetics

Biodegradation kinetics provides information about the efficacy of various nutrient supplements in soil. The values determined in this investigation were plotted in a spreadsheet and fitted to a first-order model (biodegradation kinetics). The biodegradation rate was determined by comparing it to the reaction rate constant for the first-order kinetic equation, which is given in equation:

ln[A]t=−kt+ln[A]o

where [A]_t_ is the concentration at time *t*, [A]_O_ is the concentration at time 0, and *k* is the first-order rate constant (determined by the slope of the line). 

The equation has the form of linear regression y=mx+b. Therefore, a plot of the natural log of [A] as a function of time yields a straight line. The biodegradation half-life (t½) and percentage degradation (percent D) were calculated by extrapolating the time required for TPH and PAH concentrations to decrease to half of their initial values. The R^2^ value, which is referred to as “goodness of fit” [38], revealed how well the models fit the experimental data. It is a measure of the proportion of variance in the dependent variable (TPH degradation) that can be explained by the independent variable (time).

### 4.7. Statistical Analysis

All statistical analyses were performed using a spreadsheet and R [75]. The relative growth rates of the isolates were determined using a three-parameter logistic model. A one-way analysis of variance was used to compare the mean residual total petroleum hydrocarbons and mean residual polycyclic aromatic hydrocarbons under different treatments. In all cases, the normality of variance was tested by the Shapiro–Wilk method [76], while the homogeneity of variance was tested using Levene’s test [77]. Differences were considered significant at *p* < 0.05. The *p* values were adjusted using the Holm method to control the family-wise error rate [78,79].

## 5. Conclusions

Composting is an ecofriendly method for the transformation of organic materials into biofertilizers with potential application for the remediation of petroleum-contaminated systems. Water hyacinth (WH) was found to be rich in nutrients necessary to stimulate microbial growth and activity, and in combination with spent mushroom compost (SMC) were able to enhance the degradation petroleum hydrocarbons. The organic geochemical analysis revealed the following order of biostimulatory effect for TPH biodegradation: Sterilized soil + WH (93%) > SMC + WH (89%) > NPK fertilizer (86%) > WH (82%) > SMC (75%) > Control (4%). Although the inorganic fertilizer NPK was more effective as a biostimulant than either spent mushroom compost or water hyacinth compost individually, the combination of spent mushroom and water hyacinth composts (SMC + WH) exerted greater biostimulation of crude oil degradation than NPK. These results show that a combination of different composted organic materials may prove more effective for the remediation of environmental pollutants. The 16 polycyclic aromatic hydrocarbons designated by the US EPA as priority pollutants were either completely or highly degraded in the SMC + WH treatment, indicating the potential of this amendment for the environmental remediation of soils contaminated with recalcitrant organic pollutants.

## Figures and Tables

**Table 1:** Chemical constituents of dried water hyacinth.

Parameter	Root	Stem	Leaves
Nitrogen (%)	2.0 ± 0.1 ^a^	2.3 ± 0.1 ^b^	2.8 ± 0.2 ^c^
Phosphorus (%)	4.8 ± 0.2 ^a^	5.3 ± 0.2 ^b^	7.1 ± 0.2 ^c^
Potassium (%)	6.5 ± 0.3 ^a^	8.7 ± 0.4 ^b^	12.9 ± 0.4 ^c^
TOC (%)	41.4 ± 0.5 ^b^	39.6 ± 0.2 ^a^	44.8 ± 0.4 ^c^
TOM (%)	73.2 ± 0.8 ^a^	75.7 ± 0.9 ^b^	78.2 ± 0.6 ^c^
Lignin (wt.%)	4.9 ± 0.1 ^b^	4.8 ± 0.2 ^b^	3.6 ± 0.1 ^a^
Cellulose (wt.%)	32.5 ± 0.3 ^c^	30.2 ± 0.4 ^b^	27.3 ± 0.5 ^a^
Hemicellulose (wt.%)	20.7 ± 0.3 ^a^	19.9 ± 0.3 ^a^	22.9 ± 0.2 ^b^
Reducing sugars (mg/L)	5.2 ± 0.1 ^a^	8.9 ± 0.2 ^c^	7.8 ± 0.1 ^b^
Wax (wt.%)	9.7 ± 0.2 ^a^	11.5 ± 0.3 ^b^	15.9 ± 0.4 ^c^
Total carbohydrates (wt.%)	65.4 ± 0.2 ^b^	68.2 ± 0.4 ^c^	63.6 ± 0.3 ^a^

Superscripts reflect homogenous subsets, while columns with similar superscripts are significant at *p* < 0.05.

**Table 2:** Bacterial growth during enrichment.

Day	Heterotrophic Bacterial Count (CFU/g)
5	2.92 ± 0.10 × 10^6^
10	2.60 ± 0.28 × 10^6^
15	2.10 ± 0.25 × 10^6^
20	1.87 ± 0.09 × 10^6^

**Table 3:** Characteristics of cellulase-producing isolates on carboxyl methyl cellulose plates.

Isolates	Zone of Clearance (cm)	Colony Diameter (cm)
B1	3.20	0.21
B2	1.00	0.15
B3	1.70	0.10
B4	2.30	0.23
B5	1.60	0.12
B6	1.29	0.18
B7	2.52	0.23
B8	0.50	0.15

**Table 4:** Degradation kinetics for total petroleum hydrocarbons and polycyclic aromatic hydrocarbons.

Total Petroleum Hydrocarbons					
Degradation Indices	NPK	WH	SMC	SMC + WH	Sterilized Soil + WH
Half-life (days)	19.80	22.43	27.40	17.29	15.13
Degradation (%)	85.89 ^c^	81.86 ^b^	75.16 ^a^	89.22 ^d^	92.24 ^e^
R^2^	0.992	0.984	0.971	0.998	0.996
Polycyclic Aromatic Hydrocarbons					
Half-life (days)	44.15	63.59	94.95	30.94	22.73
Degradation (%)	58.48 ^c^	45.60 ^b^	33.71 ^a^	71.54 ^d^	81.85 ^e^
R^2^	0.989	0.997	0.978	0.992	0.986

WH: water hyacinth compost; SMC: spent mushroom compost; SMC + WH: spent mushroom compost + water hyacinth compost; SS + WH: sterilized soil + water hyacinth. Different superscripts denote significantly different treatments (*p* < 0.05).

**Table 5:** Treatment setups for biostimulation-based bioremediation experiments.

Setup	Description
NPK	1000 g of polluted soil + 10% *w*/*w* NPK (20:10:10) fertilizer
WH	1000 g of polluted soil + 10% *w*/*w* water hyacinth compost
SMC	1000 g of polluted soil + 10% *w*/*w* spent mushroom compost
SMC + WH	1000 g of polluted soil + 5% *w*/*w* spent mushroom compost + 5% *w*/*w* water hyacinth compost
Sterilized soil +WH	1000 g sterilized polluted soil + 10% *w*/*w* water hyacinth compost
Control	1000 g of polluted soil

## Data Availability

The data presented in this study are available on request from the corresponding author.
